# SPAD Leaf Greenness Index: Green Mass Yield Indicator of Maize (*Zea mays* L.), Genetic and Agriculture Practice Relationship

**DOI:** 10.3390/plants10050830

**Published:** 2021-04-21

**Authors:** Piotr Szulc, Jan Bocianowski, Kamila Nowosad, Waldemar Zielewicz, Joanna Kobus-Cisowska

**Affiliations:** 1 Department of Agronomy, Poznań University of Life Sciences, Dojazd 11, 60-632 Poznań, Poland; 2 Department of Mathematical and Statistical Methods, Poznań University of Life Sciences, Wojska Polskiego 28, 60-637 Poznań, Poland; jan.bocianowski@up.poznan.pl; 3 Department of Genetics, Plant Breeding and Seed Production, Wrocław University of Environmental and Life Sciences, Grunwaldzki 24A, 53-363 Wrocław, Poland; 4 Department of Grassland and Natural Landscape Sciences, Poznań University of Life Sciences, Dojazd 11, 60-632 Poznań, Poland; waldemar.zielewicz@up.poznan.pl; 5 Department of Gastronomical Sciences and Functional Foods, Poznań University of Life Sciences, Wojska Polskiego 31, 60-624 Poznań, Poland; joanna.kobus@up.poznan.pl

**Keywords:** maize, fresh matter yield, SPAD, agriculture factors, microsatellite markers

## Abstract

The study presents the results of two field studies (Experiment I, Experiment II), whose aim was to assess the impact of agriculture factors on maize green mass and leaf greenness index (Soil and Plant Analysis Development, SPAD) in critical growth stages, as well as to determine the relationship between the SPAD index and the yield of green maize for ensiling. It was shown that thermal and humidity conditions in maize growing seasons determined the value of the SPAD leaf greenness index and the yield of maize harvested for silage. Row application of mineral fertilizer (N, NP) and selection of “stay-green” varieties guarantee a higher yield of maize green mass. Growing maize in direct sowing reduces chlorophyll content expressed in SPAD units, thereby reducing plant nitrogen condition, which significantly decreases the yield of biomass intended for silage. The SPAD leaf greenness index determined in critical stages of maize growth can be considered as a yield predictor of green mass for ensiling. The examined maize cultivars were divided into two groups on the basis of hierarchically grouping using the unweighted pair group method of arithmetic means. The first group comprised cultivars SY Cooky and Drim “stay-green,” while the second one included cultivars ES Paroli “stay-green” and ES Palazzo.

## 1. Introduction

**Figure 1:**
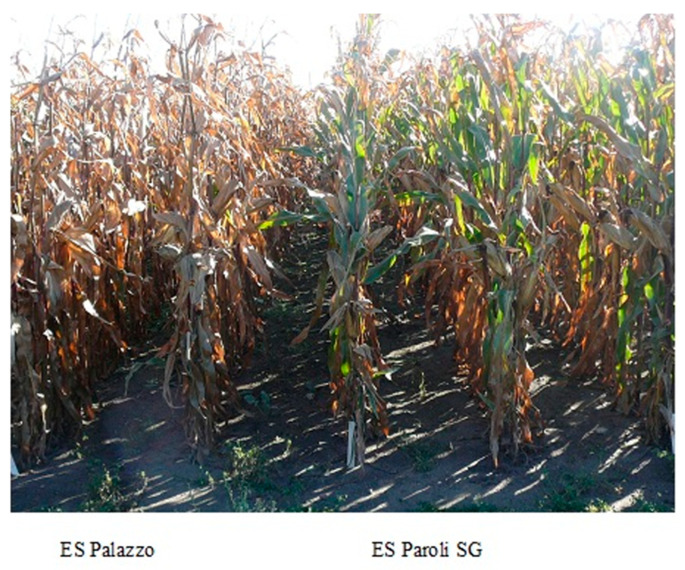
The difference in the appearance of two types of maize in adolescence. SG—effect “stay-green.”

Balanced fertilization is the basic task of sustainable agriculture [1], which should take into account all the nutrients necessary for proper growth and development [2,3]. Ensuring the optimal level of plant growth factors, including nutrient availability, guarantees the accomplishment of the yield potential [4,5]. Maize (*Zea mays* L.) is characterized by a high ability to absorb nutrients that are used in mineral fertilization [2]. Therefore, doses of mineral fertilizers should correspond to the nutritional needs, taking into account the amount of components that can be taken up from the soil [6]. Some of the reasons for low maize yield are acidic soils, insufficient nutrient supply, as well as failure to adjust the fertilization system to quantitative needs, especially the dynamics of nutrient uptake by plants throughout the growing season [3]. Periodic water deficiencies in the soil (drought), frequent in growing seasons, limit the yield potential of new, often more fertile maize hybrids [7,8]. Due to unpredictable weather conditions, the creation of hybrids with advantages in an environment with limited water resources is one of the main challenges faced by breeders [9]. “Stay-green” maize hybrids belong to such varieties. This is the result of a better developed root system [10] and a faster initial growth dynamic [11]. Hence, breeding works should be focused on greater plant health in the later growing season [12] and/or on increasing leaf greenness, known as “stay-green” [13]. This trait is an indicator of good plant health, slower aging rate (Figure 1), and drought tolerance after flowering [14]. The “stay-green” trait shows a positive correlation with plant yielding [15,16]. However, the cause-and-effect relationship between these traits and grain yield is limited by the duration of the growing season and genetic basis of the cultivar under study [17,18].

Plant yielding potential is usually assessed by comparison of cultivars grown with employment of up-to-date agronomic methods which eliminate biotic stresses (diseases, pests, weed infestation). In real terms, the correlation between genetic and agronomic progress is an important factor in influencing plant yielding potential, and cannot be attributed to neither breeding (cultivar) nor agronomy. Hence experimental researches—field experiments, aimed at assessment of cultivars depending on their type, are so important. Only such a procedure can be a determinant of progress in plant production.

The SPAD BBCH17/18 (7–8 leaf stage), SPAD BBCH63 (the beginning of pollen) and yield are the quantitative traits which are determined by large number of genes. Expression of these traits is a result of effects of environmental and quantitative trait loci (QTLs). Genetic distance between the studied hybrids influences the values of the observed traits. Simple sequence repeats (SSR) or microsatellite markers can be useful tools in taxonomy and are traditionally used for the analysis of genetic similarity/distance among genotypes.

Therefore, the aim of the conducted field study is to determine the influence of various agriculture factors on: (i) shaping green mass of maize, (ii) the SPAD index in critical stages of maize growth, (iii) determination of the relationship between the SPAD leaf greenness index and the yield of maize green mass intended for ensilage, (iv) genetic similarity between cultivars on the basis of microsatellite markers. The adopted assumptions were verified on the basis of two field experiments carried out over a period of six years.

## 2. Results

Analysis of variance indicated that the main effect of year was significant for all three traits of study in Experiment I as well as for SPAD-BBCH15/16 and yield in Experiment II (Table 1).

### 2.1. Experiment I

**Figure 2:**
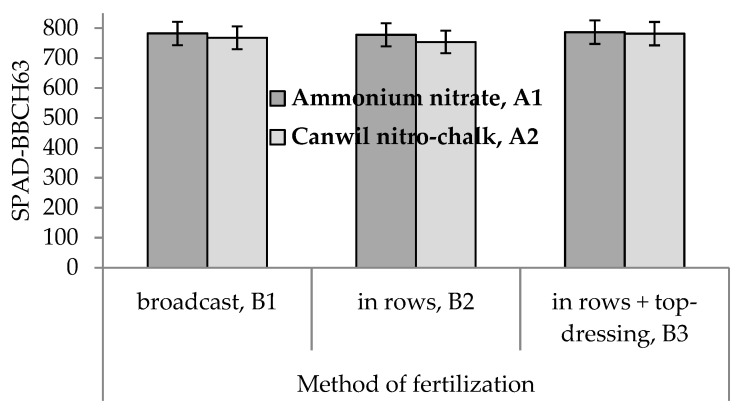
Mean values of SPAD-BBCH63 for the combinations of two types of nitrogen fertilizer and three methods of fertilization.

**Figure 3:**
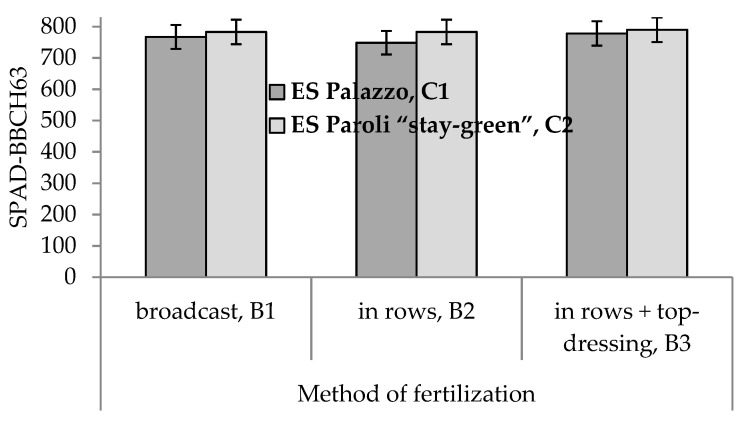
Mean values of SPAD-BBCH63 for the combinations of two cultivars and three methods of fertilization.

Analysis of variance indicated that the main effects of the method of fertilization and cultivar were statistically significant for all three traits of study in Experiment I (Table 1 and Table 2). Additionally, the main effect of type of nitrogen fertilizer was significant for SPAD-BBCH63. Type of nitrogen fertilizer × method of fertilization (Figure 2), method of fertilization × cultivar (Figure 3), and year × cultivar interactions were significant for SPAD-BBCH63. Year × cultivar interaction was significant for SPAD-BBCH17/18 (Table 1).

Mean values of SPAD-BBCH63 were larger for ammonium nitrate than for Canwil nitro-chalk in all three methods of fertilization (Figure 2).

Mean values of SPAD-BBCH63 were larger for ES Paroli “stay-green” than for ES Palazzo in all three methods of fertilization (Figure 3).

SPAD-BBCH17/18 was positively correlated with SPAD-BBCH63 in the first year of study and for all three years jointly (Table 3). Yield correlated with SPAD-BBCH17/18 and SPAD-BBCH63 in 2010 and for three years jointly. Additionally, yield was positively correlated with SPAD-BBCH63 in the second year of study (Table 3).

Multiple regression model indicated that the yield was determined by SPAD-BBCH63 in 2009 (coefficient of determination R^2^ = 17.50%) and by both SPAD-BBCH17/18 and SPAD-BBCH63 in 2010 (R^2^ = 22.00%) (Table 4).

### 2.2. Experiment II

**Figure 4:**
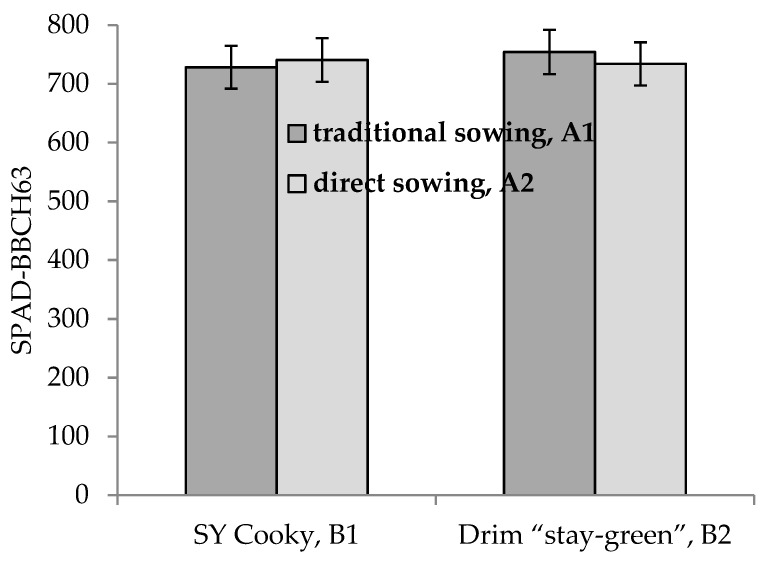
Mean values of SPAD-BBCH63 for the combinations of two cultivars and two method of sowing.

Analysis of variance indicated that the main effects of method of sowing, cultivar, method of fertilization and year × method of fertilization were statistically significant for SPAD-BBCH15/16 and yield in Experiment II (Table 1 and Table 5). Additionally, method of sowing × cultivar (Figure 4) and year × A interactions determined SPAD-BBCH63 (Table 1 and Table 5). Year × method of fertilization interaction was significant for SPAD-BBCH15/16 (Table 1).

SPAD-BBCH15/16 was positively correlated with SPAD-BBCH63 in the first year of study (*r* = 0.47) and negatively in 2014 (*r* = −0.39) (Table 6). Yield correlated positively with SPAD-BBCH15/16 in 2013, 2014, and for three years jointly and with SPAD-BBCH63 in 2013 and for three years jointly (Table 6).

Multiple regression model indicated that the yield was determined by SPAD-BBCH63 in 2013 and by SPAD-BBCH15/16 in 2014 (Table 7). Coefficients of determination for these models are equal to 38.20% and 50.80%, respectively. For all data from three years of study in obtained model *y* = −7.4 + 0.055 *x*_1_ + 0.058 *x*_2_ both SPAD-BBCH15/16 and SPAD-BBCH63 influence on yield (R^2^ = 14.80%) (Table 7).

### 2.3. Genetic Similarity of Cultivars

A total of 112 microsatellite (SSR) markers were detected with the set of 30 SSR primers. The average number of alleles per locus was 3.73, ranging from 1 to 17. The size of PCR products ranged from 63 to 267 bp. The data were computed to estimate the genetic similarity between the studied four maize cultivars based on Nei and Li’s coefficients. The highest genetic similarity (amounting to 0.652) was found between cultivars SY Cooky and Drim, whereas the lowest genetic similarity (0.394) was found for cultivars ES Palazzo and Drim (Table 8). The mean value of genetic similarity was 0.484.

The SSR markers data were used to group cultivars by the unweighted pair group method of arithmetic means (UPGMA) method. As a results of hierarchically grouping using the UPGMA, the examined maize cultivars were divided into two groups: the first group comprised cultivars SY Cooky and Drim, while the second one included cultivars ES Paroli and ES Palazzo.

Cultivars with large genetic similarity characterized the large similarity of values of all three observed traits: SPAD-BBCH15/16, SPAD-BBCH63, and yield (Table 2, Table 5, and Table 8).

## 3. Discussion

In recent years, enormous progress has been made in maize agriculture practices, and it consists of genetic progress, development of cultivation technologies, and improvement of quality parameters [19,20]. High maize for grain productions performance and versatile application possibilities result in a systematic increase in maize cultivation area in the world, which is unmatched by any other leading crop [21]. Currently, in terms of the sown area, it is second only to winter wheat (*Tricitum aestivum* L.), but it decisively dominates in terms of the quantity of harvested grain, which is also the main agricultural commodity in the world trade [21]. Allocating the field area for maize increases the efficiency of crop rotation, while a higher proportion of maize silage in the ration improves the profitability of animal feeding. Due to the deterioration of milk production profitability, it is necessary to further increase the maize cultivation for silage [22] and measures aimed at improving the quality of the produced feed [23]. Therefore, field research on the improvement of maize agrotechnics is a response to the above statement.

During the growing season, maize needs about 250 mm of rainfall in central Europe conditions. The critical period for water supply in maize is 40 days, of which 25 days fall before and 15 days after flowering. It usually occurs between 25 June and 15 August (depending on the variety). During this period, rainfall should provide 100–150 mm of water, depending on the prevailing temperatures. According to Zhao et al. [24], an increase in the average global air temperature by 1 degree Celsius reduced maize yield by 7.4%. The results obtained in the current study indicated the importance of weather conditions, mainly hydrothermal, which varied between the years of research, on the obtained green mass of maize and other crops [25,26]. The average lowest yield of maize green mass for the years was recorded in 2011 (49.17 t ha^−1^—Experiment I) and 2014 (54.28 t ha^−1^—Experiment II). These were the years characterized by the lowest total precipitation, with the highest average daily air temperature (Table 9).

Substrate moisture is one of the features that determines the nutrient supply to the developing plant root system. As reported by Qin et al. [27] and Nkebiwe et al. [28], diffusion process as a result of which the nutrient is delivered toward the root occurs faster in a moist environment than in a dry one. The results obtained in the present study clearly showed that the localized fertilization could be a method reducing the effects of periodic water shortages during the maize growing season. According to Singh et al. [29], fertilizers placed in soil with a high moisture content were more accessible to plants than those placed on the surface, which was also shown in this study. Improved efficiency of fertilizer application into the soil, compared to traditional fertilization applied on soil surface, is conditioned by the high concentration of the component in the immediate vicinity of the roots, stimulation of root system growth, and reduction of nutrient loss to the environment [30,31]. The presented results have clearly shown that row fertilization is an agriculture solution that allows for a significant increase in the size of maize biomass intended for ensilage. 

**Figure 5:**
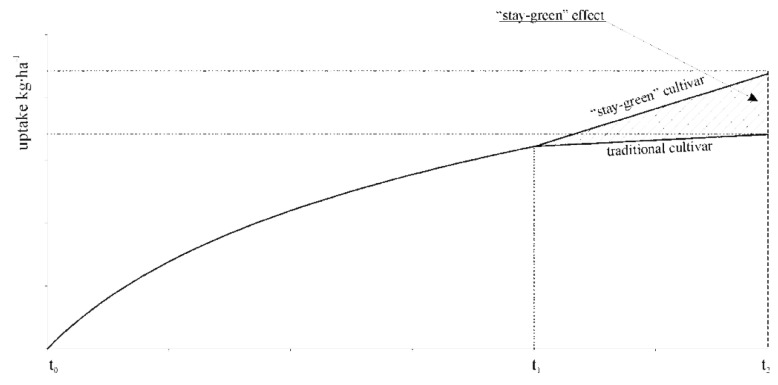
Hypothetical model of uptake of nitrogen, potassium, phosphorus, calcium, and magnesium by different types of maize cultivars [37].

Regardless of the weather conditions over the years, significantly higher green mass yield was obtained for maize sown in the cultivated soil compared to direct sowing into stubble. The difference between sowing methods was 14.11 t ha^−1^. The results of other studies [32] also indicated that the tillage method had a strong influence on maize yield. The prevailing opinion in the literature is that the use of simplified cultivation, and in particular direct sowing [33], causes a significant reduction in grain yield, maize straw yield, and protein yield [34]. It was found in this study that the “stay-green” cultivar was characterized by significantly higher maize green mass yield compared to the classic cultivar. The difference between the tested cultivars was 2.91 t ha^−1^ in Experiment I, and 4.09 t ha^−1^ in Experiment II (4.09 t ha^−1^). Szulc [35] demonstrated that in the maize vegetation period, the “stay-green” type hybrid exhibited a greater increase in dry matter of a single plant than the traditional cultivar. Also the values of absolute growth rate (AGR) of dry matter of a single plant, dry matter of leaf blades, and dry matter of grain were higher for the “stay-green” type cultivar when compared to the traditional cultivar. The differences in an increase in dry matter of a single plant grew together with cultivar generative development. The result confirms earlier reports [36]. According to this author, the second critical period of maize demand for nitrogen can be observed in the beginning of flowering. During the period from the tasseling stage (BBCH 55–59) to the stage of full silking (BBCH 65), the rate of nitrogen accumulation by maize is doubled (Figure 5). However, increased maize demand for nitrogen is short and decreases in the milk stage to the initial level, i.e., the rate of nitrogen accumulation before full flowering. According to this author, nitrogen uptake is in accordance with the course of dry matter accumulation by maize. A decrease in the rate is more gentle, and ends at the early dough stage, i.e., at dry matter content of 45%.

The result obtained in the current study was consistent with the previous report of Szulc et al. [38]. Higher grain yields obtained under row fertilization were also achieved by Mascagni and Boquet [39]. Higher yield as a result of row fertilization resulted from improved plant nutrition with nitrogen (N-NI) and phosphorus (P-NI) in the juvenile phase [40]. This was confirmed by Leach and Hameleers [41], who found that high phosphorus concentration in the dry mass of maize plants before the 6-leaf stage significantly increased maize yield. Subedi and Ma [42] also found that proper plant nutrition in the juvenile stages had a decisive effect on the final maize yield. 

In agricultural practice, a fast and non-destructive method of assessing the nitrogen nutritional status of plants is commonly applied [43,44]. It consists in determining the intensity of leaf greenness using a SPAD-502 optical device (Soil and Plant Analysis Development) or an N-Tester (Hydro) [45]. Both of these devices do not directly measure the chlorophyll content of plant leaves, but only determine the greenness index, which is the quotient of light absorption at two wavelengths, 650 and 940 nm. Rostami et al. [46] reported in a study on maize that the yield of this species was significantly linearly correlated with the SPAD index values. This linear relationship indicates that the SPAD index can serve as a tool to estimate the yield of maize biomass for silage as well as grain yields. Chlorophyll content in plant leaves is considered a predictor of yield size. It accounts for over 98% of the variation in gross primary maize production [47]. By measuring chlorophyll content, one can indirectly assess the potential absorption of infrared radiation and leaf ability to remain green [48]. The ratio of radiation absorbance at 650 nm and 940 nm can be calculated using soil plant analysis development (SPAD). The SPAD reading indicates a positive relationship with maize yield as a result of energy transport from photosynthesis due to increased production, or indicates a negative relationship with efficiency if energy is re-metabolized from chlorophyll. A study conducted by Ghimire et al. [49] clearly showed that the content of chlorophyll expressed in SPAD units was positively correlated with grain yield. Our research also demonstrated that determining the values of correlation coefficients between plant traits helps to estimate the degree of dependence, which can then be used to predict the response of fresh matter yield to the SPAD index in different phases of plant growth (statistical significant correlation coefficients between all pairs of traits for all three years of study in both experiments, except *r* = 0.07 between SPAD-BBCH15/16 and SPAD-BBCH63).

Generally, genetic divergence is not the same as phenotypic divergence but in our results we obtained association between genetic similarity of four cultivars and three observed traits: SPAD-BBCH15/16, SPAD-BBCH63, and yield. More genetic similarity was observed for cultivars from the same seed company, and less when we are comparing across companies.

## 4. Materials and Methods

### 4.1. Plant Material

Weight measurements of the whole plants were carried out during the harvest of maize for silage (black spot at the base of the kernel). On this basis, the yield of green mass of whole plants was determined (stem + leaves + ear). The dry matter content in silage (whole plants) was 38%.

For this purpose, plants from 2 m^2^ were cut out from each experimental plot, then this yield was converted into the green mass yield (t ha^−1^).

### 4.2. Field Experiments

#### 4.2.1. Experiment I

The field experiment was carried out at the Department of Agronomy of Poznań University of Life Sciences, on the fields of the Experimental and Educational Unit in Swadzim (52°26′ N; 16°45′ E) in the years 2009–2011. It was carried out as a three-factor “split-split-plot” design (random sub-block method) with four replications. The factors investigated were: A—type of nitrogen fertilizer: A1—ammonium nitrate (NH_4_NO_3_), A2—Canwil calcium ammonium nitrate (NH_4_NO_3_ + CaCO_3_ + MgCO_3_), B—fertilizer application method: B1—broadcast (the entire nitrogen dose before maize sowing), B2—row (the entire nitrogen dose simultaneously with seed sowing), (5 cm to the side and 5 cm below the seed), B3—row application complemented with top dressing [50 kg N ha^−1^ row application simultaneously with seed sowing + 50 kg N ha^−1^ for top dressing in the 5–6 leaf stage (BBCH 15/16)], and C—the type of maize cultivar: C1—ES Palazzo and C2—“stay-green” ES Paroli. The hybrids tested were from Euralis seed company. The same mineral fertilization was applied in the entire experimental field in each year of the research, prior to the experiment, in the following amount: 100 kg N ha^−1^ (fertilizer according to the 1st order factor), 80 kg P_2_O_5_ ha^−1^ (35.2 kg P ha^−1^) in the form of granulated triple superphosphate 46% P_2_O_5_, 120 kg K_2_O ha^−1^ (99.6 kg of K ha^−1^) in the form of 60% potassium salt. The assumed planting density in the years of the study was 7.95 pcs m^−2^ (79,500 seeds ha^−1^), with a spacing between rows of 70 cm and a sowing depth of 5–6 cm.

#### 4.2.2. Experiment II

The field experiment was carried out at the Department of Agronomy of Poznań University of Life Sciences, on the fields of the Experimental and Educational Unit in Swadzim (52°26′ N; 16°45′ E) in the years 2012–2014. It was carried out for three years in the same scheme in a split-split-plot design with three factors in four field replicates. The study involved the following factors: A—1st order factor—two methods of maize sowing: A1—sowing to the soil (traditional cultivation), A2—direct sowing to the stubble after winter wheat (straw harvested); B—2nd order factor—two types of cultivars: B1—traditional cultivar SY Cooky, B2—“stay-green” cultivar Drim; C—3rd order factor—2 methods of supplying NP fertilizer: C1—distributing on the entire surface before seed sowing, C2—in rows simultaneously with seed sowing. The hybrids tested were from Syngenta seed company. The same level of mineral fertilization was applied on all experimental objects in the amount of 100 kg N ha^−1^, 70 kg P_2_O_5_ ha^−1^, and 130 kg K_2_O ha^−1^. Fertilization was balanced against phosphorus, which was applied at the whole required dose in the form of ammonium phosphate under the trade name of polidap NP (18% N, 46% P_2_O_5_). N and K fertilization was performed before maize sowing using urea (46% N) and potassium salt (60%). The N dose was reduced by the amount of nitrogen present in the polidap. The assumed planting density in the years of the study was 7.95 pcs m^−2^ (79,500 seeds ha^−1^), with a spacing between rows of 70 cm and a sowing depth of 5–6 cm.

### 4.3. Weather Conditions

The characteristics of the climatic conditions during the research period were based on data from the meteorological station belonging to the Department of Agronomy, located at the Experimental and Educational Unit in Swadzim (Table 9).

#### 4.3.1. Experiment I

The highest average daily air temperature in the growing season (April–October) was recorded in 2011 (15.9 °C). In turn, the coldest growing season was in 2010 (14.5 °C). However, it should be stated in general that thermal conditions in the study years were favorable for maize growth and development. Significantly greater differences between the years of research occurred in the amount, or more precisely, the distribution of atmospheric precipitation. The highest sum for the growing season was recorded in 2010 (500.7 mm), which was higher by as much as 76.5 mm compared to the total precipitation in the 2011 growing season. It should be noted that despite the lowest precipitation in the last year of the study, as much as 218.7 mm of rain fell in July, which contributed to mild, short-term flooding of plants.

#### 4.3.2. Experiment II

Thermal conditions during maize growing in the experimental years were similar to each other and amounted on average to 15.4 °C in 2012, 15.6 °C in 2013, and 16.1 °C in the warmest year of 2014. Definitely greater differences between years occurred in the amount of precipitation. The largest sum of rainfall was recorded in 2012 (473.6 mm), which was 76.2 mm higher than the precipitation in 2013, and 121.8 mm higher compared to rainfall in 2014.

### 4.4. Soil Conditions

The field experiments (Experiment I, Experiment II) were carried out on gray-brown podzolic soil, with the grain size composition of light loamy sand shallowly deposited on light loam, belonging to the good rye soil complex. Soil abundance in basic macronutrients and soil pH in the individual years of the study are presented in Table 10. Soil samples for laboratory determinations were taken from a depth of 20 cm with a specialized auger for taking soil samples. The samples were taken in the spring, approximately 3 weeks before sowing maize. Twelve soil samples were randomly taken from the experimental plot. Their content and soil pH were assessed in accordance with the research procedure/standard (OSCHR in Poznań): P_2_O_5_—PB.64 ed. 6 from 17 October 2008; K_2_O—PB.64 ed. 6 from 17 October 2008; Mg—PB.65 ed. 6 from 17 October 2008; pH—PB.63 ed. 6 from 17 October 2008. In general, it should be stated that the plots of Experiment I had a higher soil pH compared to the plots of Experiment II (Table 10).

### 4.5. Quantitative Traits

An optical device known in Europe as Hydro N-Tester (YARA GmbH, Germany), and in the USA as SPAD-502, was used in the indirect method of determining the nutritional status of maize plants. This apparatus works by measuring the light absorption of the leaf at two wavelengths: 650 and 940 mm. The quotient of these differences is an indicator of chlorophyll content and is referred to as SPAD units (Soil and Plant Analysis Development). A high coefficient of determination (R^2^), depending on the species, was shown between the indications of the apparatus and the extracted chlorophyll quantity [50]. In Experiment I, this measurement was performed at the BBCH 17/18 stage (7–8 leaf stage) and BBCH 63 (start of pollination), while in Experiment II—BBCH 15/16 (5–6 leaf stage) and BBCH 63 (start of pollination). The mean SPAD value was derived from 25 individual measurements in each trial plot. In stage BBCH 15/16 the measurement was made on the fifth leaf, in stage BBCH 17/18 on the seventh leaf, and in stage BBCH 63 on the leaves near the ears.

### 4.6. DNA Extraction

Ten-days old maize leaves were taken from an experiment set up under laboratory conditions to extract the genomic DNA. In order to destroy the tissue structure, freezing with liquid nitrogen and grinding in TissueLyser II (Qiagen, Hilden, Germany) was used. DNA isolation was performed using a phenol extraction method. After isolation and spectrophotometric evaluation (NanoMasterGen MN-913, Syngen, Wrocław, Poland) of the quantity and quality of DNA and PCR was performed. Total of 30 primers detecting microsatellites in a maize species were used [51], available in the MaizeGDB database (www.maizegdb.org). The PCR mix contained 5.9 μL H_2_O, 1.5 μL buffer × 10 (Thermo, Waltham, USA), 2.5 μL MgCl_2_ (25 mM, Thermo), 1 μL dNTP (10 mM each, mix, Thermo), 1 μL forward (F) and reverse (R) primers (primers concentration 0.28 mM), 0.2 μL of polymerase (5 μ μL^−1^, Thermo), and genomic DNA (30 ng μL^−1^). Final reaction volume was 15 μL and was performed using a TProfessional gradient thermal cycler (Biometria GmbH, Goettingen, Germany). Amplification conditions were 94 °C for 3 min, 35 cycles at 94 °C for 30 s, 55 °C for 45 s, and 72 °C for 1 min, then final elongation at 72 °C for 10 min. Qiaxel capillary electrophoresis system (Qiagen, Hilden, Germany) was used to separate amplification products and to partially analyze the resulting SSR markers, the size of the products was determined using the ScreenGel software (Qiagen, Hilden, Germany).

### 4.7. Microsatellite Markers Analysis

On the basis of the preliminary PCR and capillary electrophoresis tests, primers with the highest degree of polymorphism were selected. All tested objects were analyzed for the presence (1) or the absence (0) of the SSR band. The obtained data were collected and constructed into a binary matrix of discrete 1-0 data.

### 4.8. Statistical Analysis

The normal distributions of the observed traits (SPAD-BBCH15/16, SPAD-BBCH63 and yield) were established by using the Shapiro–Wilk’s normality test [52]. A four-way (year, type of nitrogen fertilizer, method of fertilization and cultivar for Experiment I as well as year, method of sowing, cultivar and method of fertilization for Experiment II) analyses of variance (ANOVA) were carried out to determine the main effects and all interactions on the variability of the studied traits. Mean values and standard deviations of individual traits were calculated. Tukey’s honest significant difference (HSD) post-hoc tests were used to determine the differences across concentration for all traits according to the data model obtained from the split-split-plot experimental designed. Tukey’s HSD test is a single-step multiple comparison procedure and statistical test which can be used on raw data or in conjunction with an ANOVA (*post-hoc* analysis) to identify means that are significantly different from each other. The relationships between SPAD-BBCH15/16, SPAD-BBCH63, and yield were estimated using correlation coefficients in the study years separately and over the years. The regression analysis was used to identify the strength of the effects that the independent variables SPAD-BBCH15/16 (*x*_1_) and SPAD-BBCH63 (*x*_2_) have on a dependent variable—yield (*y*). Genetic similarity (GS) for each pair of cultivars was estimated based on the coefficient proposed by Nei and Li [53], defined as:

GS = 2N_AB_/(N_A_ + N_B_) (1)

where N_AB_ is the number of bands shared by cultivars A and B, N_A_ is the number of bands in cultivar A, and N_B_ is the number of bands in cultivar B. The cultivars were grouped using the unweighted pair group method with arithmetic mean (UPGMA).

## 5. Conclusions

Thermal and humidity conditions in maize growing seasons determine the value of the leaf greenness index (SPAD) and the yield of maize harvested for silage.

The row application of mineral fertilizer (N, NP) in maize cultivation is more effective compared to broadcast fertilization, which clearly results in obtaining a higher yield of green maize mass.

The condition for using the biological progress represented by the “stay-green” maize variety is the simultaneous recognition of yield physiology aspects and development of plant nutrition on this basis. Selection of “stay-green” varieties for silage cultivation guarantees high biomass yields for ensilage.

The row application of mineral fertilizer was better suited to the “stay-green” maize cultivars, the clear effect of which was the difference between the studied varieties in chlorophyll content expressed in SPAD units in the BBCH 63 stage (start of pollination).

The division of nitrogen fertilizer dose (ammonium nitrate, Canwil calcium ammonium nitrate) into pre-sowing row fertilization and top dressing applied at the BBCH 15/16 stage (5–6 leaf stage) increased chlorophyll content in SPAD units in the BBCH 63 stage (start of pollination) compared to the broadcast and row fertilization.

The negative effect of direct sowing on the yield of maize green mass was most likely caused by the reduced plant root system, which could be largely compensated by variety selection, and especially by row application of NP fertilizers.

The SPAD leaf greenness index (Soil and Plant Analysis Development) determined in critical stages of maize growth can be considered as a yield predictor of green mass for ensiling.

The examined maize cultivars were divided into two groups as a result of hierarchical grouping using the unweighted pair group method of arithmetic means. One of the groups comprised cultivars SY Cooky and Drim “stay-green,” while the second one: ES Paroli “stay-green” and ES Palazzo.

## Figures and Tables

**Table 1:** Mean squares from four-stratum analysis of variance (ANOVA) for SPAD-BBCH15/16, SPAD-BBCH17/18, SPAD-BBCH63 and yield in both experiments.

Scheme	Experiment I				Experiment II				
	d.f.	SPAD BBCH17/18	SPAD BBCH63	Yield		d.f.	SPAD BBCH15/16	SPAD BBCH63	Yield
Blocks	3	1833.2	6313.6	111.726	Blocks	3	2105	18,032.6	324.31
Type of nitrogen fertilizer (A)	1	314.1	7613.8 *	28.274	Method of sowing (A)	1	70,905 ***	376	4780.23 **
Residual 1	3	714.8	554.1	7.128	Residual 1	3	375	3507.4	40.15
Method of fertilization (B)	2	20,299 ***	4054 ***	18,175 *	Cultivar (B)	1	3071 *	2262	402.66 *
A × B	2	373	1098 **	8.432	A × B	1	50	6402.7 *	0.67
Residual 2	12	159.6	123.1	4.155	Residual 2	6	573	643.4	117.64
Cultivar (C)	1	32,266 ***	15,415 ***	304.08 ***	Method of fertilization (C)	1	45,023 ***	10,086	349.27 *
A × C	1	59.3	693.8	0.04	A × C	1	102	15	1.56
B × C	2	281.8	1755.2 *	2.56	B × C	1	133	1053.4	54.56
A × B × C	2	145.2	96.5	0.807	A × B × C	1	7	6	0.96
Residual 3	18	135.1	480.3	6.873	Residual 3	12	640	2174.3	50.77
Year	2	57,195 ***	4842 ***	199.1 ***	Year	2	180,953 ***	900.1	2409.57 ***
Year × A	2	24.6	1398.3	13.768	Year × A	2	47,322 ***	11,238.3 ***	761.68 ***
Year × B	4	1471.1	1060	6.951	Year × B	2	399	727	97.75
Year × C	2	8570 ***	2803 *	6.311	Year ×C	2	9487 ***	169.9	7.7
Year × A × B	4	581.1	228.3	5.454	Year × A × B	2	24	826.3	125.36
Year × A × C	2	123.5	148	2.81	Year × A × C	2	340	236.2	0.76
Year × B × C	4	338.2	218	3.122	Year × B × C	2	255	434.6	11.64
Year × A × B × C	4	133.8	232.2	3.345	Year × A × B × C	2	288	108.5	1.84
Residual 4	72	740.4	619.8	8.875	Residual 4	48	1162	862.2	41.68

d.f.—the number of degrees of freedom; *—significant at 0.05 level; **—significant at 0.01 level; ***—significant at 0.001 level.

**Table 2:** Mean values and standard deviations (s.d.) of observed traits for years, type of nitrogen fertilizer, method of fertilization and cultivars in Experiment I.

Factor	Factor’s Level	SPAD-BBCH17/18		SPAD-BBCH63		Yield (t ha^−1^)	
		Mean	s.d.	Mean	s.d.	Mean	s.d.
Year	2009	491.0 a	42.59	780.1 a	31.75	52.87 a	3.415
	2010	422.9 c	25.94	763.4 b	28.42	52.51 a	3.444
	2011	466.6 b	32.88	781.4 a	30.4	49.17 b	3.505
HSD_0.05_		13.9		12.2		1.39	
Type of nitrogen fertilizer	Ammonium nitrate, A1	461.7 a	40.12	782.3 a	24.97	51.96 a	4.001
	Canwil nitro-chalk, A2	458.7 a	48.57	767.7 b	34.91	51.07a	3.592
HSD_0.05_		14.7		10		1.25	
Method of fertilization	broadcast, B1	444.9 b	43.26	775.1 ab	30.23	51.07 a	3.707
	in rows, B2	483.6 a	39.09	765.7 b	34.09	52.22 a	3.722
	in rows + top -dressing, B3	452.1 b	41.69	784.1 a	26.4	51.26 a	3.988
HSD_0.05_		16.7		12.3		1.54	
Cultivar	ES Palazzo, C1	445.2 b	36.96	764.6 b	36.06	50.06 b	3.424
	ES Paroli “stay-green”, C2	475.2 a	46.39	785.3 a	20.79	52.97 a	3.648
HSD_0.05_		13.8		9.7		1.17	

a, b, c—in columns, means followed by the same letters are not significantly different.

**Table 3:** Correlation coefficients between SDAD-BBCH15/16, SPAD-BBCH63, and yield observed in Experiment I.

Trait	Year	SPAD-BBCH17/18	SPAD-BBCH63
SPAD-BBCH63	2009	0.48 ***	
	2010	0.28	
	2011	−0.08	
	2009–2011	0.34 ***	
Yield	2009	0.28	0.45 **
	2010	0.42 **	0.39 **
	2011	0.16	0.07
	2009-2011	0.17*	0.20 *

* *p* < 0.05; ** *p* < 0.01; *** *p* < 0.001.

**Table 4:** Regression models for identifying the strength of the effects that the independent variables SPAD-BBCH17/18 (*x*_1_) and SPAD-BBCH63 (*x*_2_) have on a dependent variable—yield (*y*) in Experiment I.

Year	Model	*p* for SPAD-BBCH17/18	*p* for SPAD-BBCH63	Percentage Variance Accounted	*F* for Model
2009	*y* = 14.9 + 0.006 *x*_1_ + 0.045 *x*_2_	0.620	0.008 **	17.50	5.99 **
2010	*y* = 6.5 + 0.045 *x*_1_ + 0.036 *x*_2_	0.016 *	0.034 *	22.00	7.64 **
2011	*y* = 33.7 + 0.018 *x*_1_ + 0.009 *x*_2_	0.260	0.593	-	0.75
2009–2011	*y* = 31.26 + 0.009 *x*_1_ + 0.021 *x*_2_	0.213	0.056	3.90	3.90 *

* *p* < 0.05; ** *p* < 0.01.

**Table 5:** Mean values and standard deviations (s.d.) of observed traits for years, methods of sowing, cultivars, and methods of fertilization in Experiment II.

Factor	Factor’s Level	SPAD-BBCH15/16		SPAD-BBCH63		Yield (t ha^−1^)	
		Mean	s.d.	Mean	s.d.	Mean	s.d.
Year	2012	590.6 a	34.45	744.3 a	44.73	71.43 a	9.29
	2013	441.1 c	48.93	733.7 a	45.47	65.13 b	12.75
	2014	529.8 b	80.08	739.8 a	43.08	54.28 c	11.77
HSD_0.05_		28.66	-	22.06	-	5.64	-
Method of sowing	traditional sowing, A1	547.7 a	76.51	737.3 a	48.09	70.67 a	9.48
	direct sowing, A2	493.3 b	83.24	741.2 a	40.31	56.56 b	12.9
HSD_0.05_		32.4	-	17.98	-	4.59	-
Cultivar	SY Cooky, B1	514.9 b	83.12	734.4 a	39.64	61.57 b	12.52
	Drim “stay-green,” B2	526.2 a	85.54	744.1 a	48.23	65.66 a	13.88
HSD_0.05_		10.03	-	17.89	-	2.36	-
Method of fertilization	broadcasting, C1	498.9 b	91.35	729.0 b	41	61.71 b	13.32
	in rows, C2	542.2 a	70.64	749.5 a	45.27	65.52 a	13.16
HSD_0.05_		33.09	-	17.51	-	3.37	-

a, b, c—in columns, means followed by the same letters are not significantly different.

**Table 6:** Correlation coefficients between SDAD-BBCH15/16, SPAD-BBCH63 and yield observed in Experiment I.

Trait	Year	SPAD-BBCH15/16	SPAD-BBCH63
SPAD-BBCH63	2012	0.47 **	
	2013	0.28	
	2014	−0.39 *	
	2012–2014	0.07	
Yield	2012	−0.02	0.07
	2013	0.39 *	0.61 ***
	2014	0.69 ***	−0.04
	2012–2014	0.36 ***	0.22 *

* *p* < 0.05; ** *p* < 0.01; *** *p*< 0.001.

**Table 7:** Regression models for identify the strength of the effects that the independent variables SPAD-BBCH15/16 (*x*_1_) and SPAD-BBCH63 (*x*_2_) have on a dependent variable—yield (*y*) in Experiment II.

Year	Model	*p* for SPAD-BBCH15/16	*p* for SPAD-BBCH63	Percentage Variance Accounted	*F* for Model
2012	*y* = 67.0 – 0.018 *x*_1_ + 0.021 *x*_2_	0.749	0.641	-	0.12
2013	*y* = −73.8 + 0.061 *x*_1_ + 0.153 *x*_2_	0.124	<0.001 ***	38.20	10.60 ***
2014	*y* = −63.0 + 0.117 *x*_1_ + 0.075 *x*_2_	<0.001 ***	0.055	50.80	16.98 ***
2012–2014	*y* = −7.4 + 0.055 *x*_1_ + 0.058 *x*_2_	< 0.001 ***	0.047 *	14.80	9.26 ***

* *p* < 0.05; ** *p* < 0.01; *** *p* < 0.001.

**Table 8:** Genetic similarity among the studied four cultivars of maize based on 112 SSR markers.

Cultivar	ES Palazzo	ES Paroli	SY Cooky	Drim
ES Palazzo	1			
ES Paroli	0.509	1		
SY Cooky	0.453	0.462	1	
Drim	0.394	0.435	0.652	1

**Table 9:** Average monthly air temperature and monthly total precipitation in vegetation seasons in Swadzim (52°26′ N; 16°45′ E).

Years	Temperature [°C]							
	IV	V	VI	VII	VIII	IX	X	Mean/Sum
Experiment I								
2009	12.9	14.0	16.0	20.3	20.1	15.8	7.6	15.2
2010	9.3	12.2	18.4	22.6	19.2	13.0	7.0	14.5
2011	12.4	15.5	19.9	18.5	19.5	15.9	9.8	15.9
Experiment II								
2012	9.3	16.3	17.0	20.0	19.8	15.0	8.6	15.4
2013	8.9	15.6	18.4	22.0	20.2	13.2	10.8	15.6
2014	11.4	14.6	17.9	23.2	18.8	16.0	11.2	16.1
Rainfalls [mm]								
Experiment I								
2009	19.2	109.9	113.8	75.4	26.2	48.6	59.2	452.3
2010	26.8	110.5	43.4	97.5	143.5	69.9	9.1	500.7
2011	9.8	22.5	66.5	218.7	50.5	28.5	27.7	424.2
Experiment II								
2012	17.4	84.4	118.1	136.2	52.7	28.4	36.4	473.6
2013	10.5	95.5	114.9	52.9	32.4	75.9	15.3	397.4
2014	50.3	80.7	44.6	51.5	56.5	39.2	29.0	351.8

**Table 10:** Soil conditions at Swadzim (52°26′ N; 16°45′ E).

Specification	Years					
	2009	2010	2011	2012	2013	2014
	Experiment I			Experiment II		
P [mg P kg^−1^ of soil]	63.1	39.0	42.2	112.0	38.0	127.0
K [mg K kg^−1^ of soil]	89.0	91.3	83.3	95.0	111.1	89.1
Mg [mg Mg kg^−1^ of soil]	42.0	37.0	44.0	28.0	23.0	36.0
pH [1 mol dm^−1^ KCl]	5.5	5.5	5.4	4.9	4.8	4.7

## Data Availability

Data available on request due to restrictions e.g., privacy or ethical. The data presented in this study are available on request from the corresponding author.
